# Influence of CAG Repeat Polymorphism on the Targets of Testosterone Action

**DOI:** 10.1155/2015/298107

**Published:** 2015-09-02

**Authors:** Giacomo Tirabassi, Angelo Cignarelli, Sebastio Perrini, Nicola delli Muti, Giorgio Furlani, Mariagrazia Gallo, Francesco Pallotti, Donatella Paoli, Francesco Giorgino, Francesco Lombardo, Loredana Gandini, Andrea Lenzi, Giancarlo Balercia

**Affiliations:** ^1^Division of Endocrinology, Department of Clinical and Molecular Sciences, Umberto I Hospital, Polytechnic University of Marche, Via Conca 71, 60126 Ancona, Italy; ^2^Department of Emergency and Organ Transplantation, Section of Internal Medicine, Endocrinology, Andrology, and Metabolic Diseases, University of Bari Aldo Moro, Piazza Giulio Cesare 11, 70124 Bari, Italy; ^3^Laboratory of Seminology-Sperm Bank, Department of Experimental Medicine, University of Rome “La Sapienza”, Viale del Policlinico 155, 00161 Rome, Italy

## Abstract

In the last decade, ample evidence has demonstrated the growing importance of androgen receptor (AR) CAG repeat polymorphism in andrology. This genetic parameter is able to condition the peripheral effects of testosterone and therefore to influence male sexual function and fertility, cardiovascular risk, body composition, bone metabolism, the risk of prostate and testicular cancer, the psychiatric status, and the onset of neurodegenerative disorders. In this review, we extensively discuss the literature data and identify a role for AR CAG repeat polymorphism in conditioning the systemic testosterone effects. In particular, our main purpose was to provide an updated text able to shed light on the many and often contradictory findings reporting an influence of CAG repeat polymorphism on the targets of testosterone action.

## 1. Introduction

Androgen receptor (AR) mediates the peripheral effects of testosterone. The main mechanism of action for AR is direct regulation of gene transcription. After the binding of an androgen to its receptor, a conformational change occurs, causing the dissociation of heat shock proteins, translocations into the nucleus, and dimerization [1]. The AR dimer binds to a specific sequence of DNA, known as a hormone response element, thereby up- or downregulating specific gene transcription. Furthermore, AR may also act by a nongenomic pathway that entails the rapid activation of kinase-signaling cascades and the modulation of intracellular calcium levels [1]. As far as the direct action is concerned, testosterone effect occurs via AR, both directly and indirectly through its metabolite, that is, dihydrotestosterone, in which it is converted by 5-*α* reductase [1].

**Figure 1:**
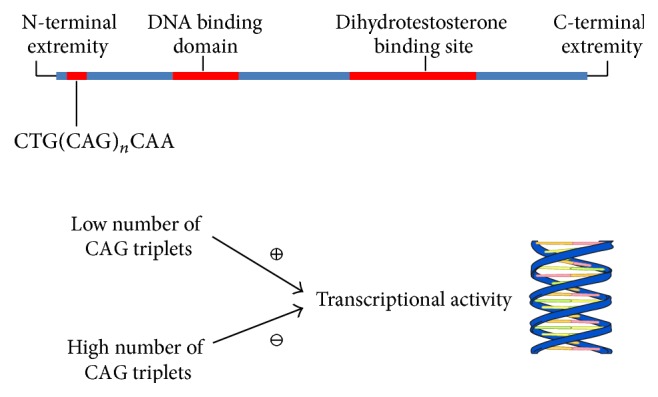
Localization of CAG repeat in androgen receptor and influence on DNA transcriptional activity. Precise mechanisms by which CAG repeat influences DNA trascription are not clear and hypotheses have been raised. Opposite association has been found between CAG repeat (polyglutamine tract) length and DNA trascriptional activity. The polyglutamine tract may indirectly affect androgen receptor function by causing structural perturbations within the transactivation domain. Alternatively, the glutamine residues may contact another protein and inhibit interactions of the activation domain with its target protein. Finally, glutamine residues could interact with a specific repressing protein, thus determining inhibition [107].

The AR gene is composed of eight exons and is located on X chromosome at q11-q12. Exon 1 of the AR gene contains a polymorphic sequence of CAG repeat, which usually varies in number from 10 to 35, and which encodes polyglutamine stretches of AR transactivation domain [2, 3] (Figure 1). Many findings suggest that CAG number is negatively correlated with the transcriptional activity of AR [4]. In fact, patients affected by Kennedy syndrome have a number of CAG repeats greater than 40 together with decreased virilization, testicular atrophy, reduced sperm production, and infertility [2]. Similarly, other studies have shown that shorter CAG repeats are associated with prostate disease, specifically cancer and benign hypertrophy, improved seminal parameters, and improved mineral bone density [2].

Ethnic differences in CAG repeat length of AR gene exist between African, Caucasian, and Asian populations. Evaluating ethnic groups is important to understand the variability of AR gene and the differences in androgen sensitivity in the general population [5]. The allele expansion in Africans was found between 18 and 20, although some African subpopulations seem to have a shorter tract [6]. In contrast, Caucasian and Asian populations have a longer CAG expansion, where the mean number of CAG repeats is, respectively, 21-22 in Caucasians [4] and 23 in Asians [7].

**Figure 2:**
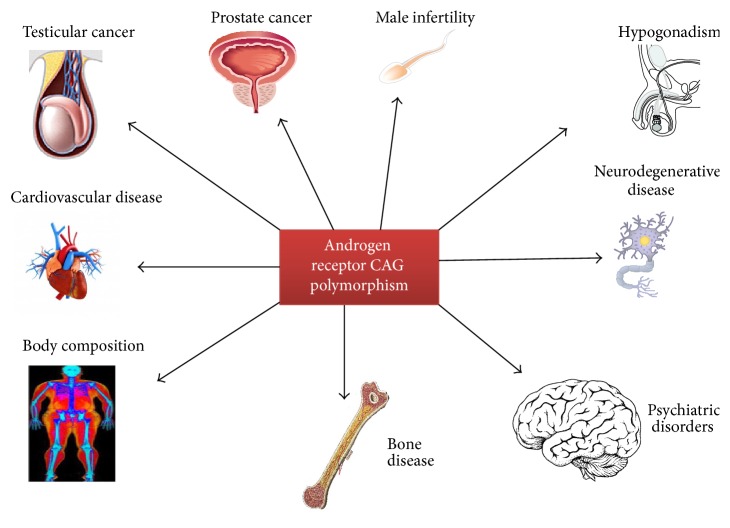
Tissues and organs influenced by the androgen receptor CAG repeat polymorphism.

In this review, we aim at discussing the literature data indicating a role for AR CAG repeat polymorphism in conditioning the peripheral effects of testosterone. In particular, our main purpose was to provide an updated contribution able to shed light on the many and often contradictory findings on the influence of CAG repeat polymorphism on the targets of testosterone action (Figure 2).

## 2. Sexual Function

The relationship between CAG repeat polymorphism and sexual function has not yet been well explored. In fact, few studies have examined this issue and knowledge on this aspect is incomplete. As far as transversal studies are concerned, a discrepancy on the role of CAG polymorphism emerges, probably due to the lack of homogeneity in recruited samples and methodological procedures. Pastuszak et al. reviewed the medical records of 85 men who presented to their clinic. AR gene CAG repeat number was found to be negatively correlated with all domains of sexual function assessable by IIEF-15 [8]. Similarly, Liu et al. carried out a free health screening in men older than 40 years and they found that, when total testosterone levels were above 3.40 ng/mL, subjects with AR CAG repeat lengths ≥ 25 had a significantly higher risk of developing andropausal symptoms (ADAM questionnaire) than those with AR CAG repeat lengths ≤ 22; interestingly, this was not observed when total testosterone levels were equal to or less than 3.40 ng/mL [9]. Conversely, Andersen et al. assessed 79 men with erectile dysfunction complaints and 340 controls in a population-based survey and they found no significant association between erectile dysfunction symptomatology and CAG repeat length [10]. However, it must be highlighted that, when evaluating erectile dysfunction complaints, these authors used a single question taken from the National Institutes of Health Consensus Development Panel on Impotence (1993) [10]. In addition, another study, conducted on 213 41–70-year-old men randomly selected from the Population Registry, reported that the CAG repeat number was positively correlated with depression, whereas men with CAG repeats higher than or equal to 23 reported decreased potency (assessed by Heinemann questionnaire) less often than the others [11].

Regarding longitudinal studies, only two reports evaluated the effects of the polymorphism in conditioning sexual function recovery after testosterone replacement therapy in hypogonadal male patients. They both found that shorter CAG length is associated with a greater improvement of several aspects of IIEF questionnaire after TRT [12, 13]. However, it must be remarked that one of these two reports evaluated a very rare form of male hypogonadism, namely, hypogonadotropic hypogonadism, and thus had a very low number of studied subjects (fifteen patients) [12]. Also, in the same report, subjects were undergoing therapy with pituitary replacement therapy and results were obtained after statistical adjustment for those confounding factors [12]. Although the other work evaluated an acceptably large sample [13], further studies will be necessary for a deeper knowledge on this important subject.

## 3. Male Infertility

Diagnostic andrology has seen considerable progress in recent years. It is now possible to establish the cause of a significant percentage of cases of infertility previously considered idiopathic. For example, it has been demonstrated that genetic factors are responsible for 15% of male infertility [14]. The correlation between male infertility and CAG repeat lengths on the AR gene is still unknown, while the link between this polymorphism and quality of spermatogenesis is even more obscure.

Numerous studies in recent years have attempted to establish the relationship between CAG repeat length variation and male infertility to find out if this variability in the AR gene could be associated with impaired spermatogenesis. Although it is now known that androgens and the AR receptor gene contribute toward correct sexual differentiation and maintenance of normal spermatogenesis, discordant results are found in the literature. In Australian, North American, and Japanese populations, an association between CAG repeat length and male infertility has been reported [15–18], but this has not been confirmed in Europe [19, 20]. Theories to justify the diverse results in different countries include the low number of samples examined, inadequate control groups, unsuitable inclusion criteria, and different ethnic origins. The latter might suggest that the association between CAG repeat length and male infertility could be valid for a given ethnic group but not necessarily significant if extended to different populations. However, other published studies demonstrated a significant correlation between CAG repeat length and male infertility regardless of ethnic origin [16].

In 2004, Ferlin et al. studied 163 infertile and 115 normozoospermic fertile men and found no significant difference in the mean number of CAG repeats between the two groups. The authors reached the same result when subdividing the 163 infertile patients by semen phenotype (45 patients with azoospermia, 87 with severe oligozoospermia, and 31 with moderate oligozoospermia). The authors found a statistically significant difference between cases and controls when analysing the combination of CAG with GGC polymorphism: infertile patients most commonly demonstrated the combination CAG = 21/GGC = 18, and very rarely CAG ≥ 23/GGC ≤ 16. Furthermore, patients with severe oligozoospermia most frequently demonstrated the combination CAG ≥ 21/GGC ≥ 18. The authors concluded that the number of CAG and GGC triplets has a combined effect on the AR receptor function and that this was the first evidence of a relationship between particular CAG/GGC haplotypes and male infertility [4].

In 2010, Castro-Nallar et al. found no statistically significant difference in the mean number of CAG repeats between 117 infertile patients (with azoospermia and oligozoospermia), of whom 93 were with idiopathic infertility and 121 normozoospermic controls. The authors suggest that the CAG triplet allele 21 might increase the risk of Sertoli cell-only syndrome, although the mechanism is not yet clear [21].

Nenonen et al., in a 2010 meta-analysis of 3915 men (1831 fertile and 2084 infertile), demonstrated that there was no statistically significant difference in the mean number of CAG repeats between the two groups. However, on dividing the CAG repeat length into three groups (i.e., CAG < 22, CAG 22-23, and CAG > 23), a binary logistic regression analysis found that men with <22 and >23 CAG repeats had an approximately 20% higher risk of being infertile than men with 22 or 23 CAG repeats [22]. These data are consistent with another 2010 report by the same authors, in which an in vitro study of CAG repeat lengths in the normal range and receptor activity did not find any inverse correlation between in vitro receptor function and the number of CAG repeats [23].

In 2013, Han et al. conducted a cytogenetic and molecular study on a population of infertile Chinese men. The authors analysed CAG polymorphism in 101 azoospermic and 54 oligozoospermic patients and 96 controls, excluding patients with Y chromosome deletions and chromosome abnormalities from the study. The authors did not find any statistically significant difference in CAG numbers between cases and controls. When analysing the joint association CAG > 23 and GGC < 23, the authors found a statistically significant difference between cases and controls only in GGC repeats [24].

It is known that the AR gene CAG repeat stretch is found in the transactivation domain of the protein that interacts with the domain by binding androgens. The fact that mutations in the CAG repeat stretch can block this interaction [25, 26] or cause changes to the protein structure demonstrates its crucial role in maintaining the function of the AR protein. This is also in agreement with the theory that AR function is at its best within the range of 10–30 CAG repeats [26] and that it could influence both protein quantity and secretion of male sexual hormones [22]. However, more studies are required to enable AR gene receptor function to be correlated with semen quality and male reproductive potential.

## 4. Prostate Disorders

The prostate is an androgen-regulated organ and androgens also play a key role in the pathogenesis of prostate cancer. In animal models the use of androgens is essential to induce prostate cancer and men and dogs castrated before puberty do not develop prostate cancer. In addition the reduction in testosterone levels is therapeutically used to slow the growth of prostate cancer [27].

It is still unclear how androgens are involved in the etiology of prostate cancer. They are able to stimulate the growth of an existing malignant process, but there is no evidence that they might trigger the cancerous process by facilitating the transformation of a benign cell into a malignant one [28].

It is likely that the genesis of prostate cancer is not induced by androgens, but that the stronger androgenic stimulation caused by receptors with shorter CAG repeats may contribute to a faster development of malignant cells. This could explain the higher risk of cancer and the earlier age of onset [6].

An experimental study showed that in prostatic epithelial cells an inverse relationship exists between AR transactivity and polyglutamine tract length [29]. Some studies have suggested that short CAG repeats constantly stimulate androgens, with increased cell proliferation and induction of somatic mutations [30, 31]. Also, short CAG repeats have been associated with more aggressive forms of prostate cancer [31, 32].

Experimental data also suggest that the increased transcriptional activity favors the formation of TMPRSS2:ERG, a fusion gene found in about 50% of prostate cancers. In the same study, which evaluated 291 men with prostate cancer (147 ERG-positive) and 1.221 cancer-free controls, subjects with shorter CAG repeats had an increased risk of ERG-positive (odds ratio (OR), 1.07 per 1 repeat decrease; 95% CI, 1.00–1.14), but not ERG-negative prostate cancer (OR, 0.99 per 1 repeat decrease; 95% CI, 0.93–1.05). These data suggest that short CAG repeats are associated with the development of TMPRSS2:ERG-positive prostate cancer [33]. On the other hand, another recent study on the Chinese population has found that shorter CAG repeat lengths were not associated with a high induction rate of TMPRSS2 and ERG proximity, a fundamental step for the TMPRSS2:ERG fusion. However, samples of 17 triplets were found more frequently in the TMPRSS2:ERG fusion positive than negative tumors and mediated a higher rate of androgen-induced TMPRSS2 and ERG colocalisation than AR with longer (24) and shorter (15) CAG repeats, suggesting that 17 triplets were associated with TMPRSS2:ERG fusion positive cancer. Also, the number of 17 triplets could have a protective role in the Chinese population which has a low frequency of TMPRSS2:ERG fusion [34].

Many authors have suggested that the CAG repeat length is inversely correlated with the risk of developing prostate cancer (Table 1). Coetzee and Ross showed for the first time that the variations of the length of the CAG are associated with prostate cancer and suggested that shorter alleles can lead to increased transactivation of androgen receptor [35]. A meta-analysis of 2004 reported that patients with prostate cancer had shorter CAG repeats and the continuous odds ratio of prostate cancer per one repeat decrement was 1.02 for CAG repeat [36]. Another meta-analysis in 2012 reported that AR CAG repeat polymorphism with ≥20 repeats might confer a protective effect among prostate cancer patients older than 45 years, but not all prostate cancer patients [37]. Interestingly, a meta-analysis of 13,346 patients and 15,172 controls from 47 reports, besides confirming that shorter CAG repeat polymorphisms of the AR are associated with the increased risk of prostate cancer, also reported that the association was not shown using prospective studies but was observed using retrospective studies. Furthermore, while the risk of prostate cancer increased predominately among Asians, this was not evident among Africans and Caucasians [38].

However, it must be acknowledged that not all studies agree in concluding that shorter CAG repeats are associated with an increased risk of cancer. While some works fail to show a statistically significant association between shorter CAG repeats and prostate cancer [39, 40], others report that shorter CAG repeats are associated with a younger age at diagnosis but not with an increased risk of disease [41]. Also, another important and recent American study showed no association between the number of CAG repeats and the risk of prostate cancer based on a continuous model [42].

Finally, shorter CAG repeats seem also to be associated with the development of benign prostatic hypertrophy (BPH). According to data from the Health Professionals Follow-Up Study, men with AR gene CAG repeat lengths of 19 or less had an OR of benign prostatic hyperplasia of 1.92 relative to men with repeat lengths of 25 or more [43]. Also, a work which examined 176 BPH patients who underwent simple prostatectomy and 41 control subjects without benign prostatic enlargement found a statistically significant (*P* < 0.02) trend for large adenoma size with short CAG repeat length among the adenoma quartiles, thus demonstrating the inverse relationship between prostatic adenoma size and AR gene CAG repeat length [44].

## 5. Cardiovascular Risk and Body Composition

The role of testosterone in the cardiovascular health of men is controversial. Some evidence exists showing that hypogonadism could be associated with a worse metabolic profile and cardiovascular risk [45]; however, more recent evidence shows that hypogonadism could represent a protective mechanism in unhealthy conditions, such as in subjects with previous cardiovascular events [46]. In this complex scenario, the potential impact of CAG repeats length of AR on cardiovascular risk and lipid profile has not been clearly established.

A recent study on 1859 men aged 20–79 years showed no direct correlation between CAG repeat length of AR and cardiometabolic risk factors [47]. Other authors also found a neutral effect of the length of AR gene polyglutamine tract on lipid levels [48, 49]. Nevertheless, even if intima media thickness of peripheral arteries, lipid parameters, insulin resistance, blood pressure, and family history of early coronary artery disease (CAD) did not differ according to AR length, shorter CAG repeat of the AR gene was found to be associated with more severe CAD [48].

On the other hand, independent associations between CAG length and adverse cardiovascular risk factors, such as high LDL [50], low HDL [51], and high blood pressure [52–54], were demonstrated by other studies; intriguingly, the association between longer CAG repeat length and low total testosterone concentrations was found to exert an adjunctive worsening effect on the metabolic profile [47, 54]. It should be finally acknowledged that the AR CAG repeat polymorphism plays a role in testosterone replacement therapy of males with hypogonadotropic hypogonadism, since shorter AR gene CAG tract length was found to yield greater metabolic improvement in response to testosterone administration [2]. Altogether, these discrepancies confirm the complexity of the role of this polymorphism in regulating the relationship between androgen effects and cardiovascular risk factors.

A possible relationship has been suggested between body composition and the CAG repeat polymorphism of the AR gene. In adolescent boys, low CAG repeat numbers in AR may be a genetic risk factor for fat accumulation [55], particularly intra-abdominal fat [52]. However, after puberty, these effects seem to disappear, possibly overruled by a strongly developing hypothalamic-pituitary-gonadal axis. Indeed, AR repeat polymorphism has little influence on absolute and relative fat mass or on its regional distribution in physically active men [56, 57]. Additionally, the AR CAG repeat length could represent a significant positive predictor, albeit modest, of lower body composition index [48] and free fat mass [58–60]. Interestingly, the number of CAG triplets was positively and significantly correlated with the decrease in abdominal fat after testosterone replacement therapy in hypogonadotropic hypogonadal men [61]. Somewhat discordant results were however reported in a cohort of 233 men with type 2 diabetes and symptoms of hypogonadism, in which shorter AR CAG was independently associated with waist circumference and body mass index [53], suggesting an effect in providing healthy anthropomorphic and metabolic features. Again, there appears to be a complex relationship between CAG repeat length and body composition, possibly influenced by genetic factors involved in type 2 diabetes, obesity, and cardiovascular disease, as well as environmental factors, including circulating total and free testosterone levels, lifestyle changes, and dietary patterns.

## 6. Bone Metabolism

The influence of CAG repeat polymorphism in the AR gene on bone health is also not clear. CAG length of the AR gene was found to have a negligible [62–64] or positive association with bone mass [65, 66]. Androgenization could certainly affect the association between AR polymorphism and bone mineral density (BMD); in 229 healthy men, the lowest age- and body mass index-adjusted average femoral neck BMD was found among men in the lowest tertile for both AR repeat length and free testosterone, whereas men in the higher categories of these variables displayed the highest BMD [66]. In contrast, Zitzmann et al. suggested that a high number of CAG repeats within the AR gene could attenuate testosterone effects on bone density and bone metabolism: the number of CAG was found to inversely and independently associate with BMD in 110 healthy men aged 20–50 years, and an increase in age-dependent bone loss in subjects with a CAG length of 22–31 compared with 14–21 CAG was reported [67]. In healthy elderly men, the AR gene CAG repeat polymorphism was shown to have a neutral effect on the determination of bone turnover and bone mineral density [68]. BMD measurements at the hip and forearm were not associated with AR CAG repeat length and there was no association of the polymorphism with any of the biochemical markers of bone turnover [68] or femoral neck BMD in older men with both normal and low BMD or history of femoral fractures [69]. Finally, rates of vertebral [65] or femoral [69] fractures in men were independent of the CAG repeats. However, while it is controversial whether CAG repeat polymorphism may affect bone metabolism under physiological conditions, shorter AR CAG tract was found to be independently associated with greater improvement of BMD in hypogonadotropic hypogonadism after treatment with testosterone replacement therapy [70]. Further studies will elucidate if normal subjects or patients with specific diseases (e.g., late onset hypogonadism, surgical hypogonadotropic hypogonadism, and Klinefelter syndrome) could take advantage of the screening of CAG repeats when evaluating testosterone replacement therapy.

## 7. Testicular Cancer

Testicular cancer (TC) accounts for 1% of all cancers in men. It comes in a broad variety of histotypes: over 90% of cases originate from the germinal epithelium of the seminiferous tubules, making this the largest group of testicular cancers. Its incidence varies from 1 per 100,000 in Asia and Africa to 9.2 per 100,000 in Denmark [71–73]. The highest incidence is in fact found in Central Europe (Denmark, Norway, and Germany) and in Caucasian populations of developed countries. As with all tumours, the aetiopathogenesis is unknown, although various predisposing factors (cryptorchidism, family history of testicular cancer, lifestyle, environmental conditions, and genetic susceptibility) have been identified. The development of testicular cancer is postulated to be due to endocrine disruption, particularly abnormalities in the action of gonadotropins and steroidal sex hormones [74]. Men with androgen insensitivity syndrome due to AR gene mutations have a higher risk of developing testicular cancer. Various recent studies have investigated the gene polymorphisms that might be involved in modulating the mechanism of action of sex hormones. Androgen insensitivity has in fact been suggested as a risk factor for testicular cancer. Epidemiological studies have demonstrated that different CAG triplet repeat lengths may play an important part in the onset of testicular cancer. Men of African origin have both fewer CAG repeats and a lower incidence of testicular cancer than do Caucasian men. However, the few published studies analysing the correlation between CAG repeat length and testicular cancer report contradictory results [75, 76].

Irvine suggested that a longer CAG repeat region could reduce the receptor's transactivation activity [77]. Many authors have tried to understand if reduced androgen sensitivity due to point mutations, or more often to an excessively long CAG repeat segment, could lead to the development of testicular dysgenesis and, consequently, increase susceptibility to testicular cancer. Meyts et al. analysed CAG repeats in a Danish population of 102 testicular cancer patients and 110 controls. No statistically significant differences in the distribution of CAG repeat number were found between the two groups, with the patients being analysed by both histotype and stage [75]. Giwercman et al. tested whether CAG plays a role in the aetiology or pathogenesis of testicular cancer in a population from Malmo consisting of 83 TC patients and 220 controls, finding no statistically significant differences in CAG repeat number between patients and the control group. However, it is interesting to note that the number of men with CAG repeat number >25 was significantly lower in seminoma patients and in seminoma + nonseminoma patients than in controls. A longer CAG repeat length was found in patients with more advanced cancer at the time of diagnosis, although this was not statistically significant. This study therefore seems to suggest that longer CAG repeat lengths may indicate a higher risk of metastasis, and it was the first to demonstrate a correlation between AR CAG repeat length, testicular germ cell cancer histology, and disease progression, albeit in a limited caseload [76].

Garolla et al. analysed 123 stage 1 testicular cancer patients against a control group of 300 fertile men studied for AR mutations, of whom 115 were selected for the CAG and GGC repeats study [78]. There were no differences in the number of CAG and GGC repeats between patients and controls. This study [78] did not confirm the differences found by Giwercman et al. in 2004 between cancer histotypes nor the greater frequency of CAG > 25 in patients than in controls [76]. Moreover, when both CAG and GGC repeats were considered together, the distribution of CAG/GGC = 20/17 was significantly higher in testicular cancer patients (8.1%) than in controls (1.7%) (*P* < 0.05) [78].

In 2012, Kristiansen et al. investigated the correlation between CAG repeat length and testicular cancer in a Norwegian population of 651 TC patients and 313 controls. No statistically significant differences were seen in the number of CAG repeats between patients and controls, even when analysed by histotype [79]. In addition, they were unable to confirm Giwercman et al.'s finding [76] that CAG > 25 was more common in patients with nonseminomatous tumours. Finally, Grassetti et al. found no significant difference in the average CAG repeat number between 302 testicular cancer patients and 322 cancer-free controls [80]. In this study, men with CAG repeat lengths below 21 or above 24 were found to have a, respectively, 50% and 76% higher risk of testicular cancer than patients with CAG 21–24. In other words, the risk of developing testicular cancer would seem to be lower for men with a CAG repeat number between 21 and 24. These results support the suggestion that normal AR function is sustained over a critical but limited range of CAG repeat numbers. Furthermore, as in Giwercman et al.'s study [76], the proportion of subjects with a long CAG repeat length (≥25) was higher in testicular cancer cases than controls; this was statistically significant for the nonseminoma group compared to controls [80]. However, other studies did not find these differences between histological groups [79].

Previous studies have correlated CAG repeat length with clinical stage of testicular cancer, reporting higher CAG repeat numbers if the tumour was advanced at diagnosis [76]. Grassetti et al. found a statistically significant difference in CAG repeat length depending on the stage of the disease, with the longest or shortest found among patients with stage II disease at the time of diagnosis [80]. In this group, the odds ratio of testicular cancer was higher for men in whom the CAG and GGC alleles were both long (CAG > 24 and GGC > 18; OR 2.65) or both short (CAG < 21 and GGC ≤ 17; OR 2.39). This trend was evident for both histotypes under study (seminoma and nonseminoma) [80].

In conclusion, a CAG repeat number of ≥25 may be considered a risk factor for the onset of testicular cancer, given its greater frequency in patients compared to controls [80]. This is of considerable scientific and oncological interest, although the underlying biological mechanism is still unclear. A greater CAG repeat number and consequent reduced efficiency of the transactivation domain may lead to a diminished AR capacity to recognize and bind androgens, making them incapable of functioning correctly and resulting in a higher concentration of free hormones. These two factors could play a part in the onset of testicular cancer. On the other hand, the least risk is seen with CAG repeat numbers between 21 and 24, the most common in the general population, thus confirming in vitro findings. Finally, stage II patients were more likely to have a CAG repeat number <21 or >24 than stage I patients [80]. These aspects lead us back to the crucial role played by the length of the polymorphic segment in AR function; a change in the number of repeats can lead to various disorders and, above all, is a risk factor for testicular cancer that should not be ignored.

The role of sex hormones in the genesis of TC has also roused considerable interest in recent years. Various studies have investigated exposure to endocrine disrupter chemicals, which may be associated with testicular cancer. Interest has focused on the synergism between gene modifications and the influence of the environment as a possible risk factor for the onset of testicular cancer. It was recently suggested that postnatal exposure might also increase the risk of developing TC and that androgen secretion during puberty might be involved in TC progression [73].

In 2008, a case-control study [81] investigated the correlation between testicular cancer and p,p-DDE, an environmental pollutant that is an androgen receptor antagonist. The authors investigated whether the risk of TC is associated with p,p′-DDE and whether this association is modified by CAG repeat polymorphisms in the AR gene. They did not find any correlation between endocrine disrupters and TC nor did they find that the risk of TC was modified by AR gene polymorphisms.

However, comparative studies of groups of single ethnic origins are still lacking, since there is a highly variable distribution of these polymorphisms in different populations worldwide. This would enable further understanding of the role of the AR gene and polymorphism frequency in the onset of testicular cancer in patients of different ethnic origins.

## 8. Psychiatric Status

Important differences in behavior, personality, and depressive tendencies exist between males and females. Men seem more inclined to search dominance [82] and to take risks [83] and are less likely to develop depression or low self-esteem compared to women [84]. It is very likely that these differences are due to different concentrations of androgens, particularly testosterone. This hormone is important in the pathogenesis of aggression [85] and in mood and self-esteem [86]. The receptor sensitivity to androgens is also of relevance. In fact, hormone sensitivity is influenced by the AR CAG polymorphism [87].

Some authors have shown an association between shorter CAG repeats and self-report measures of dominance and prestige, all of which are argued to be indices of the mating effort [88]. Others have found that men with shorter CAG repeats are those in whom the concentration of testosterone increases the most during interaction with the other sex [89]. In addition, a report by Vermeersch et al. found significant interactions between CAG repeat length and testosterone, indicating that free testosterone was more positively related to aggressive and nonaggressive risk-taking behaviour with a shorter repeat length and that an inverse association of free testosterone with depressive symptoms and a positive association with self-esteem were stronger in boys with a longer CAG repeat length. Also, free testosterone levels were found to be significantly related to dominance in boys with shorter CAG repeats, but not in those with medium and long CAG repeats [90].

Regarding depression, discordant findings exist. Some studies show an association between longer CAG repeats and higher levels of depression [91], while others find no association [92]. A relationship between testosterone levels, CAG repeat length, and depression was found in a work, which reported that, in middle-aged men, depression was significantly and inversely associated with total testosterone levels only in men with shorter CAG repeats, but not in those with the medium and long numbers of CAG repeats [93]. Even in young boys, higher ratings on sleep symptoms of depression were predicted by lower testosterone concentrations and shorter CAG lengths [94].

In addition, violent behavior [95] and impulsivity [96] have been found to be related to shorter CAG repeats, whereas other reports have denied these associations [97, 98]. Finally, it is also worth noting that several studies have shown that the presence of shorter AR CAG tracts is linked to Attention Deficit Hyperactivity Disorder and conduct disorder [99].

## 9. Neurodegenerative Disorders

CAG repeat may be involved in the pathogenesis of some neurodegenerative diseases [100]. However, the most studied and robust relationship is the one with spinal-bulbar muscular atrophy (SBMA). SBMA is an adult-onset neurodegenerative disease characterized by degeneration of the motor neuron mainly located in the spinal and bulbar regions, resulting in slowly progressive muscle weakness and atrophy [101]. SBMA patients have AR polyglutamine chain length higher than 40 as well as varying degrees of androgen insensitivity with gynecomastia, testicular atrophy, disorders of spermatogenesis, elevated serum gonadotropins, and diabetes mellitus [3, 100]. Length of CAG repeat chain is directly associated with the severity of symptoms of hyperandrogenicity and the earliness of the onset of the disease [3]. Robust evidence suggests that polyglutamine stretch exerts toxic function on neurons leading to neurological phenotypes and neurodegeneration [102]. AR is present in motoneurons as well as in other types of neurons such as gonadotropin-releasing hormone secreting cells and in hypothalamic districts controlling sexual behaviour and gonadotropin secretion [103]. Nuclear accumulation of the pathogenic AR protein has been considered to be the crucial step of neurodegenerative process, which is in turn followed by transcriptional dysregulation, axonal transport disruption, and mitochondrial dysfunction [101]. In particular, the abnormal length of polyglutamine chain induces the formation of intracellular aggregates, a clear signature of SBMA. This occurs after the association of AR with its ligand which results in the exposition of the polyglutamine tract provoking in turn an interaction with another polyglutamine AR or the production of aberrant conformational changes of AR [102]. Nuclear aggregates may sequester transcription factor coactivators essential for cell function (e.g., CREB-binding protein) [103]. However, it is worth saying that controversial results have been reported regarding the association between the amount of aggregation and neurodegeneration [104, 105].

## 10. Conclusions

In 2015, a large body of evidence indicated an important role for AR CAG polymorphism in conditioning the peripheral effect of testosterone, even if its contribution warrants further assessment because of the many controversial findings in each androgen-related action. Of note, other associations are emerging (e.g., between anogenital distance and the androgen receptor CAG repeat length [106]), but they still need further confirmation. We believe that the differing results could be justified in light of the difference in the clinical characteristics of the studied subjects, the methodology (transversal/longitudinal studies), and the number of assessed patients. Also, it must be highlighted that so far not all andrological outcomes have been analyzed in depth (e.g., sexual function). Uniformity of methodological evaluation and the study of scarcely considered outcomes are the routes that scientific research will have to take in order to clarify this important issue.

At present, AR CAG polymorphism is not recommended in the routine setting. However, in the near future, it could become of clinical relevance because of the theoretical possibility of identifying subjects more or less at risk for various disorders and more or less responsive to testosterone treatment. In this last case, study of CAG repeat length could allow us to individually tailor testosterone replacement therapy, as subjects with shorter CAG repeat could need lower doses of testosterone while men with longer repeats could require higher ones.

## Figures and Tables

**Table 1:** Summary of meta-analysis results regarding the association between androgen receptor CAG repeat and prostate cancer.

Meta-analysis	Number of studies included	Cases/controls	Opposite relation between CAG length and prostate cancer risk (yes/no)	Remarks
Zeegers et al. [36]	19	4274/5275	Yes	Modest association between shorter repeats and prostate cancer risk

Gu et al. [37]	27	2972/3792, 3835/4908, and 3372/2631 for comparisons of ≥20, 22, and 23 repeats of CAG sequence with others	Yes	AR CAG repeat polymorphism with ≥20 repeats might confer a protective effect among the prostate cancer patients older than 45 years but not all the prostate cancer patients

Sun and Lee [38]	47	13346/15172	Yes	Shorter CAG repeat sequence had an increased risk of prostate cancer (OR 1.21, 95% CI 1.10–1.34) regardless of the exact length of the CAG repeat, compared with carriers of a longer repeat sequence

OR: odds ratio; AR: androgen receptor.
